# μ-1,2-Bis­(diphenyl­phos­phino)­ethane-κ^2^ *P*:*P*′-bis­{[1,2-bis­(diphenyl­phosphino)­ethane-κ^2^ *P*,*P*′]bromidocopper(I)} acetone disolvate

**DOI:** 10.1107/S1600536808031000

**Published:** 2008-09-27

**Authors:** Wen-Juan Shi

**Affiliations:** a Jiangxi Key Laboratory of Surface Engineering, Jiangxi Science and Technology Normal University, Jiangxi 330013, People’s Republic of China

## Abstract

In the crystal structure of the title compound, [Cu_2_Br_2_(dppe)_3_]·2CH_3_COCH_3_ [dppe is 1,2-bis­(diphenyl­phosphino)­ethane, C_26_H_24_P_2_], the two Cu centers are bridged by a dppe ligand and each metal center carries one chelating dppe unit, with the fourth coordination site available for the Br^−^ anion. The mol­ecule is centrosymmetric, with the center of symmetry located between the methyl­ene C atoms of the bridging dppe ligand. The crystal structure is stabilized by intra­molecular C—H⋯Br hydrogen bonds and inter­molecular π–π inter­actions, with a centroid-to-centroid distance of 3.2055 (1) Å.

## Related literature

For related research on phosphanecopper(I) compounds as biological agents, see: Berners-Price *et al.* (1987 ▶); Goldstein *et al.* (1992 ▶); Navon *et al.* (1995 ▶). For related structures, see: Albano *et al.* (1972 ▶); Comba *et al.* (1999 ▶); Darensbourg *et al.* (1990 ▶); Eller *et al.* (1977 ▶); Leoni *et al.* (1983 ▶); Mohr *et al.* (1991 ▶); Di Nicola *et al.* (2006 ▶).
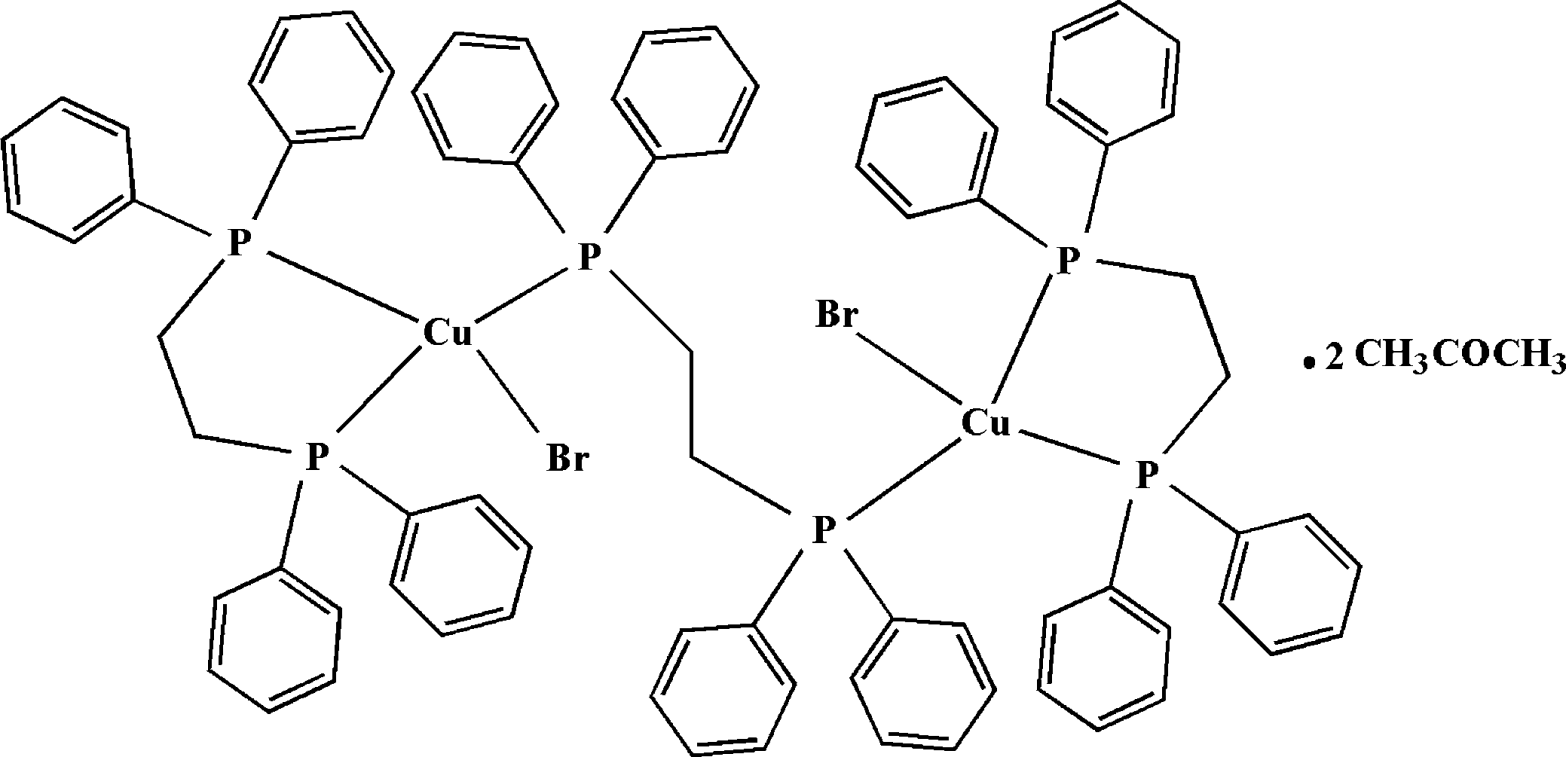


## Experimental

### 

#### Crystal data

[Cu_2_Br_2_(C_26_H_24_P_2_)_3_]·2C_3_H_6_O
*M* *_r_* = 1598.23
Monoclinic, 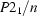

*a* = 12.5301 (6) Å
*b* = 21.8966 (10) Å
*c* = 14.8028 (7) Å
β = 105.932 (1)°
*V* = 3905.4 (3) Å^3^
*Z* = 2
Mo *K*α radiation
μ = 1.74 mm^−1^
*T* = 295 (2) K
0.20 × 0.18 × 0.17 mm

#### Data collection

Bruker SMART APEX area-detector diffractometer
Absorption correction: multi-scan (*SADABS*; Sheldrick, 1996 ▶) *T*_min_ = 0.691, *T*_max_ = 0.752
33389 measured reflections
8907 independent reflections
6429 reflections with *I* > 2σ(*I*)
*R*_int_ = 0.035

#### Refinement

*R*[*F* ^2^ > 2σ(*F* ^2^)] = 0.047
*wR*(*F* ^2^) = 0.129
*S* = 1.02
8907 reflections
435 parameters
H-atom parameters constrained
Δρ_max_ = 0.75 e Å^−3^
Δρ_min_ = −0.42 e Å^−3^

### 

Data collection: *SMART* (Bruker, 2002 ▶); cell refinement: *SAINT* (Bruker, 2002 ▶); data reduction: *SAINT*; program(s) used to solve structure: *SHELXS97* (Sheldrick, 2008 ▶); program(s) used to refine structure: *SHELXL97* (Sheldrick, 2008 ▶); molecular graphics: *SHELXTL* (Sheldrick, 2008 ▶); software used to prepare material for publication: *SHELXTL*.

## Supplementary Material

Crystal structure: contains datablocks I, global. DOI: 10.1107/S1600536808031000/sj2542sup1.cif

Structure factors: contains datablocks I. DOI: 10.1107/S1600536808031000/sj2542Isup2.hkl

Additional supplementary materials:  crystallographic information; 3D view; checkCIF report

## Figures and Tables

**Table:** 

Cu1—P3	2.2740 (8)
Cu1—P1	2.2992 (8)
Cu1—P2	2.3205 (9)
Cu1—Br1	2.4381 (5)

**Table:** 

P3—Cu1—P1	113.74 (3)
P3—Cu1—P2	122.23 (3)
P1—Cu1—P2	89.30 (3)
P3—Cu1—Br1	102.02 (3)
P1—Cu1—Br1	115.56 (3)
P2—Cu1—Br1	114.67 (3)

**Table 2:** Hydrogen-bond geometry (Å, °)

*D*—H⋯*A*	*D*—H	H⋯*A*	*D*⋯*A*	*D*—H⋯*A*
C16—H16⋯Br1	0.93	2.86	3.760 (4)	164
C32—H32⋯Br1^i^	0.93	2.82	3.666 (4)	151

Symmetry code: (i) 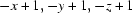.

**Fig. 1.**
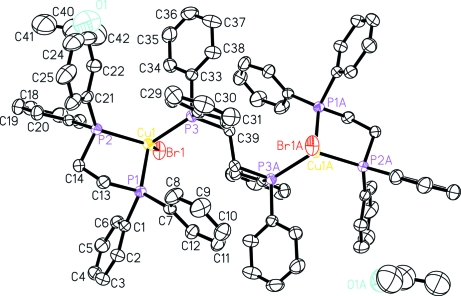
The molecular structure of (I), with displacement ellipsoids drawn at the 30% probability level. H atoms are omitted for clarity. [symmetry code: (A) -x + 1, -y + 1, -z + 1].

**Fig. 2.**
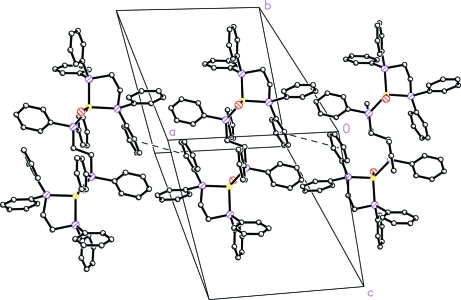
Packing diagram of the title structure showing the π–π stacking interactions.

## supplementary crystallographic information

### Comment

Detailed studies of solution equilibria and dynamics of copper(I) compounds of bidentate phosphanes have attracted considerable interest because of their potential application as potent antitumor agents (Berners-Price *et al.*, 1987) and as free radical scavengers in industrial processes (Goldstein *et al.*, 1992; Navon *et al.*, 1995). Some mononuclear (Darensbourg *et al.*, 1990; Leoni *et al.*, 1983) and dinuclear phosphanecopper(I) compounds (Eller *et al.*, 1977; Mohr *et al.*, 1991) with coordinated and bridging halide anions and with phosphane ligands in various coordination modes have been isolated and characterized. In this work, 1,2-bis(diphenylphosphino)ethane (dppe) was adopted as a ligand which coordinates to the copper(I) ions in both bridging and chelating modes.

The asymmetric unit of the title compound (Fig. 1) consists of one half of the centrosymmetric dinuclear molecule Cu_2_Br_2_(dppe)_3_ and an acetone solvate molecule. In the molecule Cu_2_Br_2_(dppe)_3_, each copper(I) center adopts a distorted tetrahedral geometry due to the constraint imposed by a chelating dppe ligand with a P(1)—Cu(1)—P(2) angle of 89.30 (3)°, which is comparable to what has been observed in other similar structures (Albano *et al.*, 1972; Comba *et al.*, 1999). The copper(I)–phosphane distances are also in the range expected from other known structures (Albano *et al.*, 1972; Comba *et al.*, 1999; Di Nicola *et al.*, 2006).

The title compound can be stablized by intramolecular C—H···Br hydrogen bonds between Br(1)^-^ anions and –CH groups from phenyl rings. Additionally, the structure is held intact through intermolecular π–π stacking interactions [centroid-to-centroid distance of 3.2055 (1) Å], displaying a one-dimensional supramolecular array (Fig. 2).

### Experimental

1,2-Bis(diphenylphosphino)ethane (40 mg, 0.1 mmol) was added to an acetone suspension (7 ml) of CuBr (10 mg, 0.07 mmol). After the addition, a precipitate slowly formed and the suspension was stirred for 12 h. The precipitate was filtered off and the resulting colorless filtrate was allowed to cool in a refrigerator. Colorless block shaped crystals were obtained after two weeks. Yield: 10 mg (20%).

### Refinement

All H-atoms were positioned geometrically and refined using a riding model with d(C—H) = 0.93 Å or 0.97 Å, *U*_iso_ = 1.2*U*_eq_(C) for aromatic and methylene H atoms; 0.96 Å, *U*_iso_ = 1.5*U*_eq_(C) for CH_3_ groups.

### Crystal data

**Table:**

[Cu_2_Br_2_(C_26_H_24_P_2_)_3_]·2C_3_H_6_O	*F*(000) = 1644
*M**_r_* = 1598.23	*D*_x_ = 1.359 Mg m^−^^3^
Monoclinic, *P*2_1_/*n*	Mo *K*α radiation, λ = 0.71073 Å
Hall symbol: -P 2yn	Cell parameters from 6062 reflections
*a* = 12.5301 (6) Å	θ = 2.3–23.5°
*b* = 21.8966 (10) Å	µ = 1.74 mm^−^^1^
*c* = 14.8028 (7) Å	*T* = 295 K
β = 105.932 (1)°	Block, colourless
*V* = 3905.4 (3) Å^3^	0.20 × 0.18 × 0.17 mm
*Z* = 2	

### Data collection

**Table:**

Bruker SMART APEX area-detector diffractometer	8907 independent reflections
Radiation source: fine-focus sealed tube	6429 reflections with *I* > 2σ(*I*)
graphite	*R*_int_ = 0.035
φ and ω scans	θ_max_ = 27.5°, θ_min_ = 1.9°
Absorption correction: multi-scan (SADABS; Sheldrick, 1996)	*h* = −16→16
*T*_min_ = 0.691, *T*_max_ = 0.752	*k* = −28→28
33389 measured reflections	*l* = −19→19

### Refinement

**Table:**

Refinement on *F*^2^	Primary atom site location: structure-invariant direct methods
Least-squares matrix: full	Secondary atom site location: difference Fourier map
*R*[*F*^2^ > 2σ(*F*^2^)] = 0.047	Hydrogen site location: inferred from neighbouring sites
*wR*(*F*^2^) = 0.129	H-atom parameters constrained
*S* = 1.02	*w* = 1/[σ^2^(*F*_o_^2^) + (0.0643*P*)^2^ + 1.6786*P*] where *P* = (*F*_o_^2^ + 2*F*_c_^2^)/3
8907 reflections	(Δ/σ)_max_ = 0.001
435 parameters	Δρ_max_ = 0.75 e Å^−^^3^
0 restraints	Δρ_min_ = −0.42 e Å^−^^3^

### Special details

**Table:**

Geometry. All e.s.d.'s (except the e.s.d. in the dihedral angle between two l.s. planes) are estimated using the full covariance matrix. The cell e.s.d.'s are taken into account individually in the estimation of e.s.d.'s in distances, angles and torsion angles; correlations between e.s.d.'s in cell parameters are only used when they are defined by crystal symmetry. An approximate (isotropic) treatment of cell e.s.d.'s is used for estimating e.s.d.'s involving l.s. planes.
Refinement. Refinement of *F*^2^ against ALL reflections. The weighted *R*-factor *wR* and goodness of fit *S* are based on *F*^2^, conventional *R*-factors *R* are based on *F*, with *F* set to zero for negative *F*^2^. The threshold expression of *F*^2^ > σ(*F*^2^) is used only for calculating *R*-factors(gt) *etc*. and is not relevant to the choice of reflections for refinement. *R*-factors based on *F*^2^ are statistically about twice as large as those based on *F*, and *R*- factors based on ALL data will be even larger.

### Fractional atomic coordinates and isotropic or equivalent isotropic displacement parameters (Å^2^)

**Table:**

	*x*	*y*	*z*	*U*_iso_*/*U*_eq_	
Cu1	0.37525 (3)	0.569117 (17)	0.27963 (2)	0.04207 (12)	
Br1	0.43216 (4)	0.472630 (18)	0.22494 (3)	0.06925 (14)	
P1	0.19903 (6)	0.57078 (4)	0.29823 (5)	0.04037 (18)	
P2	0.32268 (6)	0.64379 (4)	0.16474 (5)	0.04127 (18)	
P3	0.51111 (6)	0.58486 (4)	0.41525 (5)	0.04102 (19)	
O1	0.6704 (6)	0.6982 (3)	0.0724 (5)	0.200 (2)	
C1	0.0907 (3)	0.52128 (14)	0.2279 (2)	0.0460 (7)	
C2	−0.0179 (3)	0.52408 (17)	0.2345 (3)	0.0589 (9)	
H2	−0.0345	0.5479	0.2809	0.071*	
C3	−0.1007 (3)	0.4917 (2)	0.1726 (3)	0.0726 (11)	
H3	−0.1730	0.4937	0.1776	0.087*	
C4	−0.0775 (4)	0.4568 (2)	0.1038 (3)	0.0771 (12)	
H4	−0.1342	0.4356	0.0617	0.093*	
C5	0.0292 (4)	0.4530 (2)	0.0968 (3)	0.0753 (12)	
H5	0.0451	0.4290	0.0503	0.090*	
C6	0.1137 (3)	0.48532 (16)	0.1596 (2)	0.0571 (9)	
H6	0.1862	0.4825	0.1552	0.069*	
C7	0.1730 (2)	0.57326 (15)	0.4131 (2)	0.0462 (7)	
C8	0.1871 (4)	0.6258 (2)	0.4651 (3)	0.0743 (12)	
H8	0.2035	0.6620	0.4388	0.089*	
C9	0.1774 (4)	0.6263 (2)	0.5561 (3)	0.0931 (14)	
H9	0.1868	0.6625	0.5902	0.112*	
C10	0.1542 (4)	0.5734 (2)	0.5954 (3)	0.0840 (13)	
H10	0.1466	0.5737	0.6562	0.101*	
C11	0.1421 (4)	0.5207 (2)	0.5464 (3)	0.0732 (11)	
H11	0.1280	0.4846	0.5741	0.088*	
C12	0.1507 (3)	0.52019 (17)	0.4549 (3)	0.0583 (9)	
H12	0.1414	0.4837	0.4215	0.070*	
C13	0.1466 (3)	0.64480 (15)	0.2445 (2)	0.0500 (8)	
H13A	0.0672	0.6476	0.2358	0.060*	
H13B	0.1822	0.6780	0.2850	0.060*	
C14	0.1726 (2)	0.64918 (15)	0.1491 (2)	0.0475 (7)	
H14A	0.1453	0.6877	0.1193	0.057*	
H14B	0.1352	0.6164	0.1085	0.057*	
C15	0.3318 (3)	0.63429 (15)	0.0446 (2)	0.0470 (7)	
C16	0.3772 (3)	0.58138 (18)	0.0207 (3)	0.0615 (9)	
H16	0.4025	0.5511	0.0655	0.074*	
C17	0.3851 (4)	0.5733 (2)	−0.0706 (3)	0.0768 (12)	
H17	0.4163	0.5376	−0.0860	0.092*	
C18	0.3486 (4)	0.6159 (3)	−0.1366 (3)	0.0796 (13)	
H18	0.3539	0.6097	−0.1974	0.096*	
C19	0.3035 (4)	0.6686 (2)	−0.1138 (3)	0.0823 (13)	
H19	0.2784	0.6984	−0.1596	0.099*	
C20	0.2945 (3)	0.67856 (19)	−0.0235 (2)	0.0662 (10)	
H20	0.2637	0.7146	−0.0090	0.079*	
C21	0.3690 (3)	0.72282 (16)	0.1894 (2)	0.0531 (8)	
C22	0.4791 (3)	0.7350 (2)	0.1940 (3)	0.0744 (11)	
H22	0.5254	0.7040	0.1840	0.089*	
C23	0.5198 (5)	0.7950 (3)	0.2142 (4)	0.1008 (16)	
H23	0.5935	0.8036	0.2176	0.121*	
C24	0.4521 (6)	0.8405 (3)	0.2287 (4)	0.1076 (17)	
H24	0.4797	0.8800	0.2412	0.129*	
C25	0.3452 (5)	0.8287 (2)	0.2252 (3)	0.0970 (15)	
H25	0.2997	0.8597	0.2364	0.116*	
C26	0.3033 (4)	0.76971 (17)	0.2049 (3)	0.0718 (10)	
H26	0.2294	0.7620	0.2017	0.086*	
C27	0.4849 (3)	0.64567 (15)	0.4905 (2)	0.0475 (7)	
C28	0.4577 (3)	0.70269 (16)	0.4509 (3)	0.0602 (9)	
H28	0.4553	0.7085	0.3881	0.072*	
C29	0.4343 (4)	0.75082 (19)	0.5011 (3)	0.0774 (12)	
H29	0.4174	0.7889	0.4728	0.093*	
C30	0.4358 (4)	0.7428 (2)	0.5926 (4)	0.0809 (13)	
H30	0.4183	0.7752	0.6267	0.097*	
C31	0.4630 (4)	0.6877 (2)	0.6339 (3)	0.0831 (13)	
H31	0.4651	0.6825	0.6967	0.100*	
C32	0.4880 (3)	0.63863 (18)	0.5832 (3)	0.0651 (10)	
H32	0.5068	0.6010	0.6124	0.078*	
C33	0.6459 (2)	0.60902 (15)	0.4006 (2)	0.0472 (7)	
C34	0.6647 (3)	0.6054 (2)	0.3137 (3)	0.0666 (10)	
H34	0.6106	0.5895	0.2630	0.080*	
C35	0.7655 (4)	0.6258 (2)	0.3016 (3)	0.0855 (14)	
H35	0.7780	0.6238	0.2426	0.103*	
C36	0.8450 (4)	0.6485 (2)	0.3751 (4)	0.0830 (13)	
H36	0.9117	0.6620	0.3662	0.100*	
C37	0.8283 (3)	0.65166 (19)	0.4613 (4)	0.0785 (12)	
H37	0.8838	0.6667	0.5118	0.094*	
C38	0.7281 (3)	0.63241 (17)	0.4743 (3)	0.0627 (9)	
H38	0.7162	0.6353	0.5335	0.075*	
C39	0.5496 (2)	0.51720 (14)	0.4915 (2)	0.0455 (7)	
H39A	0.5914	0.4894	0.4632	0.055*	
H39B	0.5979	0.5300	0.5516	0.055*	
C40	0.6595 (9)	0.6699 (4)	0.0016 (8)	0.188 (2)	
C41	0.6260 (8)	0.6948 (4)	−0.0902 (6)	0.193 (2)	
H41A	0.5854	0.7319	−0.0894	0.289*	
H41B	0.5795	0.6661	−0.1320	0.289*	
H41C	0.6903	0.7034	−0.1113	0.289*	
C42	0.7128 (8)	0.6120 (4)	0.0087 (7)	0.198 (3)	
H42A	0.7886	0.6160	0.0453	0.297*	
H42B	0.7104	0.5975	−0.0530	0.297*	
H42C	0.6752	0.5835	0.0387	0.297*	

### Atomic displacement parameters (Å^2^)

**Table:**

	*U*^11^	*U*^22^	*U*^33^	*U*^12^	*U*^13^	*U*^23^
Cu1	0.0368 (2)	0.0466 (2)	0.0328 (2)	0.00363 (15)	0.00805 (15)	−0.00058 (15)
Br1	0.0828 (3)	0.0659 (3)	0.0499 (2)	0.0296 (2)	0.0208 (2)	−0.00313 (17)
P1	0.0385 (4)	0.0433 (4)	0.0407 (4)	0.0002 (3)	0.0131 (3)	0.0015 (3)
P2	0.0408 (4)	0.0452 (4)	0.0372 (4)	−0.0033 (3)	0.0097 (3)	0.0021 (3)
P3	0.0370 (4)	0.0487 (5)	0.0363 (4)	0.0002 (3)	0.0081 (3)	0.0015 (3)
O1	0.242 (5)	0.196 (5)	0.182 (4)	−0.043 (4)	0.090 (4)	−0.045 (4)
C1	0.0458 (17)	0.0483 (18)	0.0425 (17)	−0.0066 (14)	0.0096 (14)	0.0054 (14)
C2	0.0463 (19)	0.067 (2)	0.062 (2)	−0.0061 (16)	0.0114 (16)	0.0013 (18)
C3	0.050 (2)	0.085 (3)	0.076 (3)	−0.015 (2)	0.0053 (19)	0.014 (2)
C4	0.074 (3)	0.083 (3)	0.060 (2)	−0.031 (2)	−0.005 (2)	0.002 (2)
C5	0.089 (3)	0.076 (3)	0.058 (2)	−0.023 (2)	0.014 (2)	−0.012 (2)
C6	0.059 (2)	0.062 (2)	0.051 (2)	−0.0112 (17)	0.0167 (17)	0.0006 (17)
C7	0.0359 (15)	0.059 (2)	0.0459 (17)	0.0012 (13)	0.0149 (13)	0.0025 (15)
C8	0.103 (3)	0.070 (3)	0.064 (2)	−0.018 (2)	0.046 (2)	−0.012 (2)
C9	0.124 (4)	0.097 (3)	0.071 (3)	−0.023 (3)	0.048 (3)	−0.029 (3)
C10	0.092 (3)	0.120 (4)	0.050 (2)	−0.010 (3)	0.036 (2)	−0.002 (2)
C11	0.078 (3)	0.085 (3)	0.062 (2)	0.004 (2)	0.029 (2)	0.021 (2)
C12	0.063 (2)	0.059 (2)	0.056 (2)	0.0033 (17)	0.0222 (17)	0.0058 (17)
C13	0.0466 (17)	0.0470 (18)	0.060 (2)	0.0072 (14)	0.0207 (15)	0.0049 (15)
C14	0.0435 (16)	0.0478 (18)	0.0487 (18)	0.0021 (13)	0.0084 (14)	0.0085 (14)
C15	0.0441 (17)	0.058 (2)	0.0376 (16)	−0.0107 (15)	0.0095 (13)	0.0012 (14)
C16	0.063 (2)	0.074 (2)	0.051 (2)	0.0039 (19)	0.0222 (17)	−0.0006 (18)
C17	0.081 (3)	0.099 (3)	0.060 (2)	−0.008 (2)	0.036 (2)	−0.013 (2)
C18	0.081 (3)	0.116 (4)	0.045 (2)	−0.023 (3)	0.023 (2)	−0.008 (2)
C19	0.088 (3)	0.110 (4)	0.045 (2)	−0.014 (3)	0.012 (2)	0.020 (2)
C20	0.075 (3)	0.072 (3)	0.046 (2)	−0.009 (2)	0.0075 (17)	0.0068 (18)
C21	0.067 (2)	0.0541 (19)	0.0369 (16)	−0.0173 (16)	0.0125 (15)	0.0035 (14)
C22	0.070 (2)	0.085 (3)	0.062 (2)	−0.029 (2)	0.0084 (19)	0.006 (2)
C23	0.099 (3)	0.113 (4)	0.084 (3)	−0.058 (3)	0.014 (3)	0.005 (3)
C24	0.152 (5)	0.083 (3)	0.087 (3)	−0.051 (3)	0.032 (3)	−0.019 (3)
C25	0.154 (4)	0.064 (3)	0.083 (3)	−0.020 (3)	0.049 (3)	−0.012 (2)
C26	0.105 (3)	0.050 (2)	0.065 (2)	−0.012 (2)	0.032 (2)	−0.0030 (18)
C27	0.0428 (17)	0.0547 (19)	0.0439 (17)	−0.0046 (14)	0.0100 (14)	−0.0057 (14)
C28	0.068 (2)	0.058 (2)	0.051 (2)	0.0054 (18)	0.0112 (17)	−0.0041 (17)
C29	0.080 (3)	0.058 (2)	0.087 (3)	0.006 (2)	0.012 (2)	−0.011 (2)
C30	0.076 (3)	0.080 (3)	0.090 (3)	−0.008 (2)	0.028 (2)	−0.037 (3)
C31	0.092 (3)	0.105 (4)	0.058 (2)	−0.016 (3)	0.031 (2)	−0.026 (3)
C32	0.082 (3)	0.066 (2)	0.049 (2)	−0.005 (2)	0.0206 (19)	−0.0041 (17)
C33	0.0408 (16)	0.0500 (18)	0.0513 (18)	0.0015 (14)	0.0135 (14)	0.0051 (15)
C34	0.049 (2)	0.099 (3)	0.054 (2)	0.0012 (19)	0.0168 (17)	0.007 (2)
C35	0.068 (3)	0.121 (4)	0.078 (3)	0.010 (3)	0.038 (2)	0.025 (3)
C36	0.057 (2)	0.085 (3)	0.115 (4)	−0.006 (2)	0.038 (3)	0.014 (3)
C37	0.054 (2)	0.075 (3)	0.107 (4)	−0.0156 (19)	0.023 (2)	−0.022 (3)
C38	0.054 (2)	0.065 (2)	0.072 (2)	−0.0098 (17)	0.0218 (18)	−0.0135 (19)
C39	0.0381 (15)	0.0523 (18)	0.0436 (17)	0.0000 (13)	0.0069 (13)	0.0058 (14)
C40	0.221 (5)	0.172 (5)	0.169 (4)	−0.058 (4)	0.052 (4)	−0.042 (4)
C41	0.213 (5)	0.174 (5)	0.176 (4)	−0.071 (4)	0.028 (5)	−0.032 (4)
C42	0.233 (6)	0.178 (5)	0.167 (5)	−0.041 (4)	0.029 (5)	−0.048 (4)

### Geometric parameters (Å, °)

**Table:**

Cu1—P3	2.2740 (8)	C18—H18	0.9300
Cu1—P1	2.2992 (8)	C19—C20	1.389 (6)
Cu1—P2	2.3205 (9)	C19—H19	0.9300
Cu1—Br1	2.4381 (5)	C20—H20	0.9300
P1—C7	1.818 (3)	C21—C26	1.374 (5)
P1—C1	1.824 (3)	C21—C22	1.388 (5)
P1—C13	1.845 (3)	C22—C23	1.412 (6)
P2—C15	1.824 (3)	C22—H22	0.9300
P2—C21	1.830 (3)	C23—C24	1.363 (8)
P2—C14	1.834 (3)	C23—H23	0.9300
P3—C27	1.823 (3)	C24—C25	1.352 (7)
P3—C33	1.838 (3)	C24—H24	0.9300
P3—C39	1.845 (3)	C25—C26	1.395 (6)
O1—C40	1.193 (11)	C25—H25	0.9300
C1—C6	1.373 (5)	C26—H26	0.9300
C1—C2	1.391 (5)	C27—C32	1.371 (5)
C2—C3	1.377 (5)	C27—C28	1.381 (5)
C2—H2	0.9300	C28—C29	1.366 (5)
C3—C4	1.367 (6)	C28—H28	0.9300
C3—H3	0.9300	C29—C30	1.362 (6)
C4—C5	1.372 (6)	C29—H29	0.9300
C4—H4	0.9300	C30—C31	1.354 (6)
C5—C6	1.395 (5)	C30—H30	0.9300
C5—H5	0.9300	C31—C32	1.395 (6)
C6—H6	0.9300	C31—H31	0.9300
C7—C8	1.369 (5)	C32—H32	0.9300
C7—C12	1.381 (5)	C33—C34	1.372 (5)
C8—C9	1.385 (6)	C33—C38	1.377 (5)
C8—H8	0.9300	C34—C35	1.396 (5)
C9—C10	1.362 (6)	C34—H34	0.9300
C9—H9	0.9300	C35—C36	1.352 (7)
C10—C11	1.349 (6)	C35—H35	0.9300
C10—H10	0.9300	C36—C37	1.351 (7)
C11—C12	1.387 (5)	C36—H36	0.9300
C11—H11	0.9300	C37—C38	1.387 (5)
C12—H12	0.9300	C37—H37	0.9300
C13—C14	1.537 (5)	C38—H38	0.9300
C13—H13A	0.9700	C39—C39^i^	1.533 (6)
C13—H13B	0.9700	C39—H39A	0.9700
C14—H14A	0.9700	C39—H39B	0.9700
C14—H14B	0.9700	C40—C41	1.417 (9)
C15—C16	1.379 (5)	C40—C42	1.422 (9)
C15—C20	1.385 (5)	C41—H41A	0.9600
C16—C17	1.394 (5)	C41—H41B	0.9600
C16—H16	0.9300	C41—H41C	0.9600
C17—C18	1.337 (6)	C42—H42A	0.9600
C17—H17	0.9300	C42—H42B	0.9600
C18—C19	1.368 (7)	C42—H42C	0.9600
			
P3—Cu1—P1	113.74 (3)	C19—C18—H18	120.3
P3—Cu1—P2	122.23 (3)	C18—C19—C20	121.2 (4)
P1—Cu1—P2	89.30 (3)	C18—C19—H19	119.4
P3—Cu1—Br1	102.02 (3)	C20—C19—H19	119.4
P1—Cu1—Br1	115.56 (3)	C15—C20—C19	119.4 (4)
P2—Cu1—Br1	114.67 (3)	C15—C20—H20	120.3
C7—P1—C1	104.76 (14)	C19—C20—H20	120.3
C7—P1—C13	104.07 (15)	C26—C21—C22	118.8 (4)
C1—P1—C13	98.90 (15)	C26—C21—P2	124.6 (3)
C7—P1—Cu1	122.50 (10)	C22—C21—P2	116.6 (3)
C1—P1—Cu1	120.75 (11)	C21—C22—C23	119.0 (5)
C13—P1—Cu1	101.66 (10)	C21—C22—H22	120.5
C15—P2—C21	101.58 (15)	C23—C22—H22	120.5
C15—P2—C14	102.75 (14)	C24—C23—C22	120.6 (5)
C21—P2—C14	102.84 (16)	C24—C23—H23	119.7
C15—P2—Cu1	123.55 (11)	C22—C23—H23	119.7
C21—P2—Cu1	120.64 (11)	C25—C24—C23	120.5 (5)
C14—P2—Cu1	102.29 (10)	C25—C24—H24	119.8
C27—P3—C33	100.83 (15)	C23—C24—H24	119.8
C27—P3—C39	105.88 (15)	C24—C25—C26	119.7 (5)
C33—P3—C39	102.06 (14)	C24—C25—H25	120.1
C27—P3—Cu1	115.67 (11)	C26—C25—H25	120.1
C33—P3—Cu1	115.37 (11)	C21—C26—C25	121.4 (5)
C39—P3—Cu1	115.16 (10)	C21—C26—H26	119.3
C6—C1—C2	119.0 (3)	C25—C26—H26	119.3
C6—C1—P1	119.1 (3)	C32—C27—C28	117.5 (3)
C2—C1—P1	121.5 (3)	C32—C27—P3	124.7 (3)
C3—C2—C1	120.2 (4)	C28—C27—P3	117.8 (3)
C3—C2—H2	119.9	C29—C28—C27	122.1 (4)
C1—C2—H2	119.9	C29—C28—H28	119.0
C4—C3—C2	120.5 (4)	C27—C28—H28	119.0
C4—C3—H3	119.7	C30—C29—C28	119.7 (4)
C2—C3—H3	119.7	C30—C29—H29	120.2
C3—C4—C5	120.1 (4)	C28—C29—H29	120.2
C3—C4—H4	120.0	C31—C30—C29	119.8 (4)
C5—C4—H4	120.0	C31—C30—H30	120.1
C4—C5—C6	119.8 (4)	C29—C30—H30	120.1
C4—C5—H5	120.1	C30—C31—C32	120.7 (4)
C6—C5—H5	120.1	C30—C31—H31	119.7
C1—C6—C5	120.4 (4)	C32—C31—H31	119.7
C1—C6—H6	119.8	C27—C32—C31	120.2 (4)
C5—C6—H6	119.8	C27—C32—H32	119.9
C8—C7—C12	117.8 (3)	C31—C32—H32	119.9
C8—C7—P1	121.5 (3)	C34—C33—C38	118.7 (3)
C12—C7—P1	120.3 (3)	C34—C33—P3	119.7 (3)
C7—C8—C9	121.4 (4)	C38—C33—P3	121.7 (3)
C7—C8—H8	119.3	C33—C34—C35	119.8 (4)
C9—C8—H8	119.3	C33—C34—H34	120.1
C10—C9—C8	119.7 (4)	C35—C34—H34	120.1
C10—C9—H9	120.2	C36—C35—C34	120.4 (4)
C8—C9—H9	120.2	C36—C35—H35	119.8
C11—C10—C9	120.1 (4)	C34—C35—H35	119.8
C11—C10—H10	119.9	C37—C36—C35	120.6 (4)
C9—C10—H10	119.9	C37—C36—H36	119.7
C10—C11—C12	120.3 (4)	C35—C36—H36	119.7
C10—C11—H11	119.8	C36—C37—C38	119.7 (4)
C12—C11—H11	119.8	C36—C37—H37	120.1
C7—C12—C11	120.6 (4)	C38—C37—H37	120.1
C7—C12—H12	119.7	C33—C38—C37	120.8 (4)
C11—C12—H12	119.7	C33—C38—H38	119.6
C14—C13—P1	108.1 (2)	C37—C38—H38	119.6
C14—C13—H13A	110.1	C39^i^—C39—P3	114.0 (3)
P1—C13—H13A	110.1	C39^i^—C39—H39A	108.7
C14—C13—H13B	110.1	P3—C39—H39A	108.7
P1—C13—H13B	110.1	C39^i^—C39—H39B	108.7
H13A—C13—H13B	108.4	P3—C39—H39B	108.7
C13—C14—P2	110.4 (2)	H39A—C39—H39B	107.6
C13—C14—H14A	109.6	O1—C40—C41	125.0 (10)
P2—C14—H14A	109.6	O1—C40—C42	117.3 (10)
C13—C14—H14B	109.6	C41—C40—C42	115.1 (11)
P2—C14—H14B	109.6	C40—C41—H41A	109.5
H14A—C14—H14B	108.1	C40—C41—H41B	109.5
C16—C15—C20	118.8 (3)	H41A—C41—H41B	109.5
C16—C15—P2	119.1 (3)	C40—C41—H41C	109.5
C20—C15—P2	122.1 (3)	H41A—C41—H41C	109.5
C15—C16—C17	120.0 (4)	H41B—C41—H41C	109.5
C15—C16—H16	120.0	C40—C42—H42A	109.5
C17—C16—H16	120.0	C40—C42—H42B	109.5
C18—C17—C16	121.2 (4)	H42A—C42—H42B	109.5
C18—C17—H17	119.4	C40—C42—H42C	109.5
C16—C17—H17	119.4	H42A—C42—H42C	109.5
C17—C18—C19	119.4 (4)	H42B—C42—H42C	109.5
C17—C18—H18	120.3		
			
P3—Cu1—P1—C7	−8.49 (14)	Cu1—P2—C14—C13	39.9 (2)
P2—Cu1—P1—C7	−133.75 (13)	C21—P2—C15—C16	136.2 (3)
Br1—Cu1—P1—C7	109.07 (13)	C14—P2—C15—C16	−117.6 (3)
P3—Cu1—P1—C1	−145.43 (12)	Cu1—P2—C15—C16	−3.3 (3)
P2—Cu1—P1—C1	89.31 (12)	C21—P2—C15—C20	−43.9 (3)
Br1—Cu1—P1—C1	−27.88 (12)	C14—P2—C15—C20	62.3 (3)
P3—Cu1—P1—C13	106.71 (12)	Cu1—P2—C15—C20	176.7 (2)
P2—Cu1—P1—C13	−18.55 (12)	C20—C15—C16—C17	0.2 (5)
Br1—Cu1—P1—C13	−135.73 (12)	P2—C15—C16—C17	−179.9 (3)
P3—Cu1—P2—C15	119.84 (12)	C15—C16—C17—C18	−0.5 (6)
P1—Cu1—P2—C15	−122.24 (12)	C16—C17—C18—C19	0.5 (7)
Br1—Cu1—P2—C15	−4.25 (12)	C17—C18—C19—C20	−0.3 (7)
P3—Cu1—P2—C21	−12.44 (15)	C16—C15—C20—C19	0.1 (5)
P1—Cu1—P2—C21	105.48 (14)	P2—C15—C20—C19	−179.9 (3)
Br1—Cu1—P2—C21	−136.53 (14)	C18—C19—C20—C15	0.0 (6)
P3—Cu1—P2—C14	−125.61 (11)	C15—P2—C21—C26	114.5 (3)
P1—Cu1—P2—C14	−7.68 (11)	C14—P2—C21—C26	8.4 (3)
Br1—Cu1—P2—C14	110.30 (11)	Cu1—P2—C21—C26	−104.5 (3)
P1—Cu1—P3—C27	−43.39 (12)	C15—P2—C21—C22	−66.6 (3)
P2—Cu1—P3—C27	61.77 (13)	C14—P2—C21—C22	−172.8 (3)
Br1—Cu1—P3—C27	−168.53 (12)	Cu1—P2—C21—C22	74.3 (3)
P1—Cu1—P3—C33	−160.69 (12)	C26—C21—C22—C23	−0.2 (5)
P2—Cu1—P3—C33	−55.52 (12)	P2—C21—C22—C23	−179.1 (3)
Br1—Cu1—P3—C33	74.17 (12)	C21—C22—C23—C24	−0.1 (7)
P1—Cu1—P3—C39	80.75 (12)	C22—C23—C24—C25	0.8 (8)
P2—Cu1—P3—C39	−174.08 (11)	C23—C24—C25—C26	−1.2 (8)
Br1—Cu1—P3—C39	−44.39 (12)	C22—C21—C26—C25	−0.2 (6)
C7—P1—C1—C6	−147.2 (3)	P2—C21—C26—C25	178.6 (3)
C13—P1—C1—C6	105.6 (3)	C24—C25—C26—C21	0.9 (7)
Cu1—P1—C1—C6	−3.7 (3)	C33—P3—C27—C32	−109.5 (3)
C7—P1—C1—C2	40.1 (3)	C39—P3—C27—C32	−3.5 (3)
C13—P1—C1—C2	−67.1 (3)	Cu1—P3—C27—C32	125.4 (3)
Cu1—P1—C1—C2	−176.4 (2)	C33—P3—C27—C28	71.9 (3)
C6—C1—C2—C3	−0.8 (5)	C39—P3—C27—C28	177.9 (3)
P1—C1—C2—C3	171.9 (3)	Cu1—P3—C27—C28	−53.3 (3)
C1—C2—C3—C4	−0.3 (6)	C32—C27—C28—C29	−0.1 (5)
C2—C3—C4—C5	0.9 (7)	P3—C27—C28—C29	178.6 (3)
C3—C4—C5—C6	−0.5 (7)	C27—C28—C29—C30	−1.0 (6)
C2—C1—C6—C5	1.2 (5)	C28—C29—C30—C31	1.5 (7)
P1—C1—C6—C5	−171.7 (3)	C29—C30—C31—C32	−0.9 (7)
C4—C5—C6—C1	−0.6 (6)	C28—C27—C32—C31	0.7 (5)
C1—P1—C7—C8	−141.9 (3)	P3—C27—C32—C31	−177.9 (3)
C13—P1—C7—C8	−38.5 (3)	C30—C31—C32—C27	−0.2 (7)
Cu1—P1—C7—C8	75.5 (3)	C27—P3—C33—C34	−137.3 (3)
C1—P1—C7—C12	45.7 (3)	C39—P3—C33—C34	113.6 (3)
C13—P1—C7—C12	149.0 (3)	Cu1—P3—C33—C34	−12.0 (3)
Cu1—P1—C7—C12	−97.0 (3)	C27—P3—C33—C38	40.8 (3)
C12—C7—C8—C9	−1.2 (6)	C39—P3—C33—C38	−68.2 (3)
P1—C7—C8—C9	−173.8 (4)	Cu1—P3—C33—C38	166.2 (3)
C7—C8—C9—C10	0.5 (8)	C38—C33—C34—C35	−0.5 (6)
C8—C9—C10—C11	0.9 (8)	P3—C33—C34—C35	177.7 (3)
C9—C10—C11—C12	−1.5 (7)	C33—C34—C35—C36	0.7 (7)
C8—C7—C12—C11	0.6 (5)	C34—C35—C36—C37	0.1 (8)
P1—C7—C12—C11	173.3 (3)	C35—C36—C37—C38	−1.0 (7)
C10—C11—C12—C7	0.8 (6)	C34—C33—C38—C37	−0.4 (6)
C7—P1—C13—C14	176.0 (2)	P3—C33—C38—C37	−178.5 (3)
C1—P1—C13—C14	−76.3 (2)	C36—C37—C38—C33	1.2 (7)
Cu1—P1—C13—C14	47.8 (2)	C27—P3—C39—C39^i^	80.6 (3)
P1—C13—C14—P2	−59.6 (3)	C33—P3—C39—C39^i^	−174.3 (3)
C15—P2—C14—C13	168.9 (2)	Cu1—P3—C39—C39^i^	−48.5 (4)
C21—P2—C14—C13	−85.9 (2)		

Symmetry codes: (i) −*x*+1, −*y*+1, −*z*+1.

### Hydrogen-bond geometry (Å, °)

**Table:**

*D*—H···*A*	*D*—H	H···*A*	*D*···*A*	*D*—H···*A*
C16—H16···Br1	0.93	2.86	3.760 (4)	164.
C32—H32···Br1^i^	0.93	2.82	3.666 (4)	151.

Symmetry codes: (i) −*x*+1, −*y*+1, −*z*+1.
